# Study of breast implants mammography examinations for identification of suitable image quality criteria

**DOI:** 10.1186/s13244-019-0816-5

**Published:** 2020-01-03

**Authors:** Cláudia Sá dos Reis, Isabelle Gremion, Nicole Richli Meystre

**Affiliations:** 1 School of Health Sciences (HESAV), University of Applied Sciences and Arts Western Switzerland (HES-SO), Av. de Beaumont 21, 1011 Lausanne, Switzerland; 2 Discipline of Medical Radiation Sciences, School of Molecular and Life Sciences, Curtin University, GPO Box U1987, Perth, Western Australia 6845 Australia; 3 CISP - Centro de Investigação em Saúde Pública, Escola Nacional de Saúde Pública, Universidade NOVA de Lisboa, Lisbon, Portugal

**Keywords:** Radiographers practice, Breast compression, Breast positioning, Prostheses, Eklund

## Abstract

**Purpose:**

To characterise the mammography technique used in breast cancer screening programmes for breast implants (BI) and to identify if the image quality (IQ) criteria available in literature are applicable to BI imaging.

**Methods:**

The study was conducted in two phases: literature review to find IQ criteria used in mammography combining keywords in several sources; and assessment of 1207 BI mammograms using the criteria that was identified previously to see if they were achieved or not. An observation grid was used to collect information about positioning, beam energy, compression force, and exposure mode. Descriptive statistics and Student’s *t* test and χ^2^ test were performed according to the nature of the variables.

**Results:**

Forty-seven out of 2188 documents were included in the analysis, with 13 items identified to assess the quality of positioning, 4 for sharpness, 3 for artefacts, and 2 for exposure parameters. After applying the criteria to BI mammograms, retroglandular fat was not included in 37.3% of the images. The “Pectoral-Nipple-Line” criterion was achieved in 35% of MLO/ML images. The placement of the implant (subpectoral/subglandular) or performing the Eklund had significant influence on the visible anatomy (*p* = < 0.005), alongside whether the breast was aligned to the detector’s centre.

**Conclusions:**

Some of the criteria used to assess standard mammograms were not applicable to BI due to implant overlap. The alignment of the image with the detector’s centre seems to have an impact on the amount of visible tissue. Further studies are necessary to define the appropriate protocol, technique, and suitable quality criteria to assess BI mammograms.

## Key points

- Lack of harmonised practice to acquire breast implant mammography examinations
- IQ criteria for standard mammography are not always applicable for breast implants
- Specific criteria for image quality assessment of breast implant mammograms are necessary
- Implant location has impact on image quality
- Eklund Manoeuvre is important for a better visualisation of breast tissue

## Introduction

Breast augmentation is a commonly performed procedure either for cosmetics reasons or for reconstruction purpose following mastectomy. Prosthesis implantation is one of the most common techniques in use and familiarity with the necessary image acquisition techniques and imaging appearances helps to perform and assess the examinations to gain a better diagnosis [1, 2]. Mammography is one of imaging modalities that can be used to assess certain types of prosthesis (saline, silicone) and under certain circumstances (women over 40s as initial examination or search for extra capsular rupture) [3]. However, available guidelines and studies [4–8] do not present detailed guidance about breast implants (BI) mammography technique and/or image quality (IQ) criteria regarding the assessment of breast positioning, contrast, artefacts, noise levels, and sharpness as is provided for routine mammography examinations. This lack of guidance impacts on radiographers’ practice as they evaluate the image quality, even with the obvious anatomical changes during the regular BI mammography examinations. This may lead to suboptimal examinations, impacting image quality and radiation dose [9, 10]. Some studies showed a wide variation in practice highlighting the importance to identify the most appropriate approach to better diagnose breast pathologies in this specific context [10, 11].

In Switzerland, women with breast implants are invited by the regional breast cancer screening programmes (BCSP) to have a mammogram and that promotes discussion about IQ assessment protocols, namely the criteria to use, the importance of each criteria and the number of projections to be performed.

This study aims to identify the mammography techniques used to image breast implants in 14 Swiss institutions and to evaluate image quality to verify which available criteria are suitable for this specific context.

## Methods

The study was conducted in two phases, one dedicated to a literature review to identify current practice in mammography imaging of breast implants, especially criteria that should be used to evaluate the IQ. The second phase was based on the assessment of BI mammography examinations to verify if the criteria identified during phase one are applicable or not to this context.

### Literature review

BI imaging literature review was conducted to identify protocols, technical aspects, and criteria for IQ assessment of mammography images. Sources used in this phase included scientific databases (Medline, PubMed, ScienceDirect, Scopus, SpringerLink, Wiley Online Library), organisations of national healthcare systems (hospitals, regulatory bodies, breast screening programmes), and international agencies (EUSOBI and EUREF). Only documents published in English, Spanish, German, or French were considered for comparability issues, as other languages were not mastered by the team. The keywords combinations must be found either in the title or abstract. The keywords searched were mammography AND breast implants AND radiographers; mammography AND criteria OR image quality OR positioning; Eklund AND mammography OR breast implant; Breast implant AND mammography OR positioning; Image quality AND Breast Implant OR Eklund OR mammography; Image quality AND Eklund OR technique. These keywords were used in the same combinations in all databases above mentioned. A manual search was also conducted to include guidelines and recommendations provided by organisations of national healthcare systems (hospitals, regulatory bodies, etc.), professional colleges (e.g. American College of Radiology [ACR], Royal College of Radiologists [RCR]), and scientific associations. Inclusion criteria to select studies or guidelines were focused in mammography, breast positioning, techniques and protocols, quality control, and clinical IQ. Imaging modalities to study BI were also included, namely advantages and limitations of each modality in comparison to mammography and also the available guidelines promoted by organisations and agencies. Qualitative and quantitative peer-reviewed studies, research and recommendations published after 1990 were included and the documents identified were reviewed and compared.

### Analysis of breast mammography implants

**Fig. 1:**
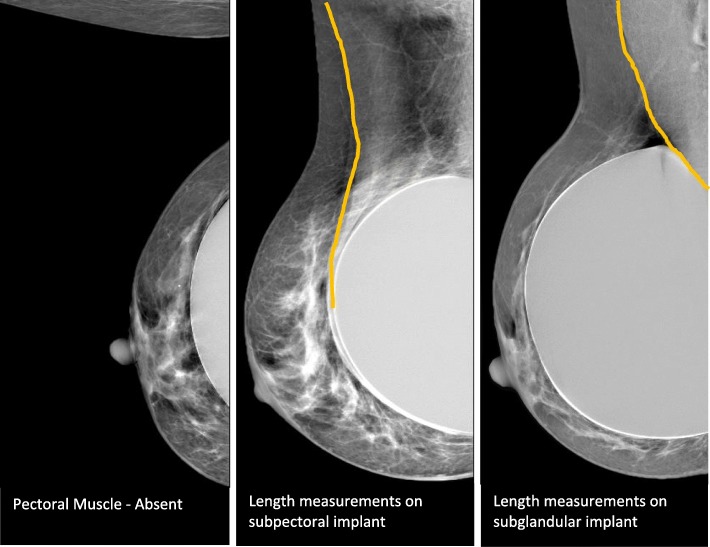
Strategy to measure the length of pectoral muscle

The second phase of this study was based on the IQ evaluation of mammographic images acquired in 14 institutions providing breast cancer screening programmes (BCSP) within two out of nine regional BCSP available in Switzerland, two in Vaud (VD), and twelve in Geneva (GVA). Examinations included were those considering patients with BI that attended at the invitation of the screening programme during 2016. Examinations that were incomplete or had major artefacts (i.e. pacemaker) were excluded. For each examination included in the assessment, the following data was collected:

- General examination data (state where exam was performed, date)
- Position of implant (subglandular, subpectoral)
- Patient’s age
- Projections performed [craniocaudal (CC), mediolateral oblique (MLO), mediolateral (ML)]
- Technique (beam energy, and with or without Eklund Manœuvre to push back the implant)
- Acquisition mode (manual or automatic)
- Compression force
- Breast thickness
- Muscle Length (in MLO and ML projections) (Fig. 1).

For the muscle length measurements [12], the “1-cm rule” was not adopted because different breast compressions were applied for both techniques (Eklund, no Eklund). CC was performed with Eklund and MLO was performed without Eklund. That has an impact on the compression force performed during the examination and, consequently, it can promote a different spread of breast tissue. Eklund can allow a stronger compression and the amount of glandular tissue visible can be higher, making the comparison between both projection CC and MLO not fair.

The alignment of the breast was also analysed. To assess if the breast was correctly centred and aligned with the detector, the observers visually evaluated the distance between the centre of the breast and the limits of detector. Laterally for CC and inferior/superior for ML/MLO images. If the distance was similar in both ways, it was considered centred.

The equipment available to acquire the images was from three manufacturers: General Electric, Hologic, and Philips Microdose. All units had a quality control programme in place to ensure the exams were performed according to the required standards of each local breast cancer screening centre and also according to manufacturer specifications. Data was available in digital imaging and communications in medicine (DICOM) headers and others were collected using the viewer system of each institution. Three qualified diagnostic radiographers assessed the same set of images according to the criteria available for positioning, artefacts, sharpness, exposure, and breast implants identified from the literature review (phase 1). They were classified as present, partially present, or absent depending on the nature of each criterion. The observers did not share the workload, and when a difference on the classification was observed between observers, a consensus was reached between the participants to classify the criterion. For this phase, a LCD monitor (EIZO Radiforce G31) calibrated according to DICOM greyscale standard display function (GSDF) standard was used for image display to simulate the environment that radiographers had in BCSP. Room lighting was dimmed and constant during the assessment task. Approval was obtained from participant institutions and from the Ethical Board of Swiss Ethics Committees on Research.

The statistical analysis was performed using SPSS and Excel software to perform descriptive statistics, Student’s *t* test and *χ*^2^ test. The criteria presented on Table 1 were all analysed considering the impact of positioning (CC, MLO, ML), implant location (subglandular/subpectoral) and technique (compression, alignment, Eklund) on the visible anatomical details and image quality. It was chosen to present and discuss only the image quality criteria that had statistical significant difference (*p* < 0.05).

**Table 1:** Criteria to assess mammography examinations to be used by radiographers before sending the examination to radiologists for *CC*, craniocaudal; *MLO*, mediolateral oblique; and *ML*, mediolateral images

Criteria	References	CC	MLO	ML	Type
Breast centrally placed	[4, 5, 13, 14]	X	X	X	Positioning (13)
Presence of pectoral muscle (PM)	[8, 13, 14]	X			
Pectoral muscle visualised down to the level of PNL	[6, 8, 13–15]		X	X	
Visualisation of retroglandular adipose tissue	[8, 13, 14]	X	X	X	
Medial border of the breast included on the image	[6, 8, 13, 14]	X			
Axillary tail demonstrated	[6, 8, 13–15]	X			
Superior breast edge included	[13]		X	X	
Inferior breast edge included	[14]		X	X	
Full visualisation of inferior breast tissue	[14]		X	X	
Inframammary angle clearly demonstrated	[6, 8, 13–16]		X	X	
Nipple in profile or transected by skin	[6, 8, 13, 14, 16]	X	X	X	
Nipple in the midline (+/− 10°)	[8, 14, 16]	X			
Symmetrical mirror images R/L images	[6, 8, 13–15]	X	X	X	
No skin folds	[6, 13–15]	X	X	X	Artefacts (3)
No artefacts	[6, 14, 15]	X	X	X	
Skin edges visualised	[13]	X	X	X	
Spread of breast tissue to differentiate adipose from fibroglandular tissue	[4, 5] [16]	X	X	X	Sharpness (4)
Sharpness of glandular tissue	[6, 13, 14, 16]	X	X	X	
Sharpness of vascular structures	[5, 13, 16]	X	X	X	
Visually sharp reproduction of skin structure (rosettes from pores)	[13]	X	X	X	
Good penetration of thicker areas without over penetration of thin areas	[6, 14, 16]	X	X	X	Parameters (2)
Appropriate contrast	[5, 6, 14, 16]	X	X	X	

## Results

This section is organised into two sessions, one presenting the IQ criteria identified during literature review and the other on the image criteria analysis of BI mammograms.

### Literature review

A total of 2188 studies were obtained using the combination of keywords and databases presented above. From those, 2020 were excluded after title reading because they were either repeated or were irrelevant to the topic (no BI imaging, or no information about mammography, or the documents were not fully available). Additionally, ten documents presenting relevant guidance/recommendation were found by manual search in databases related to international agencies (National BCSP, DONNA, EUSOBI, EU, EUREF, NHSBSP) and added to the analysis [6, 8, 13, 14, 16–21]. Abstracts of 98 papers were read and 61 were excluded due to the lack of relevance for this study reporting treatment options, or exploring other medical imaging modalities that were not the focus of this search. Thirty-seven papers from the database search were finally included in this review due to relevant information provided regarding mammographic technique (compression, exposure parameters, projections that should be used and how many) [2, 10, 22–25]; criteria to assess mammographic exam [4, 5, 21, 26–28] and discussion about the role of BI mammography (advantages, limitations, and difficulties) [1–3, 9, 11, 19, 24, 25, 29–43].

From the literature, it is noted that mammography can be used for breast cancer screening of women with breast implants even when is challenging to detect pathologies with the implant superimposed on the breast tissue [2, 24, 25, 33, 44]. Silicone BI affect image quality of mammograms, causing low detectability because the silicone is dense and it can obscure anatomical details and pathologies, depending on its size and thickness, and also depending on the X-ray beam characteristics [9]. The recommendation from the American College of Radiology (ACR) [3] is to select mammography or breast tomosynthesis to study implants when patients are over 40s, and if a clinical examination is equivocal for implant rupture. To evaluate saline BI when the patient is asymptomatic, as initial imaging for any age, mammography or tomosynthesis is not recommended.

When performed, mammography brings also challenges in compression and in performing the Eklund Manœuvre (implant is pushed back to reduce its superimposition on breast tissue). According to the literature, radiographers are afraid of causing ruptures due to lack of guidance about when to stop compression and also due to the type and/or the lack of training for this specific situation [10, 45].

Criteria to assess mammography images available in the literature are mainly for mammograms without implants [4, 6, 15, 21, 27, 46, 47] and are related to specific anatomical details that should be included in the images (pectoral muscle, inframammary angle, retroglandular adipose tissue). Sharpness, artefacts, information/labeling, and exposure parameters were also parameters identified in the review that should be assessed to decide if the image is diagnostic.

A summary of parameters adapted from the literature for the three main projection used for imaging BI is presented in Table 1. Positioning encompasses the group of criteria with more items to assess (13), followed by sharpness (4), artefacts (3), and exposure parameters (2). Possible scales that can be used, namely PGMI (Perfect, good, moderate, inadequate), are also available in the literature and it is possible to verify there is a wide range of options available in clinical practice and even within scales [4, 27].

Regarding specifically BI mammography, the recommendation from National Health Service Breast Screening Program (NHSBCSP) from the United Kingdom (UK) [19], is to visualise the breasts (size, shape, contour, nipple) before and after the exam and record all relevant information. Positioning is also highlighted and it should be performed in a way that allows the demonstration of maximum breast tissue. Eklund views are recommended to demonstrate the anterior breast tissue with the implant displaced posteriorly. These guidelines also noted that if the implant is immobile (encapsulated), a true lateral view (ML) may be considered as an alternative even though the real value of this projection is not evident in the literature.

### Analysis of breast implants mammography images

This group of results is based on the application of the criteria identified above (Table 1) to assess 1207 images produced by 14 institutions that belong to GVA BCSP (52%) and to VD BCSP (48%). The average age of patients with BI participating in the BCSP was 54 ± 4.4 ranging between 50 and 74 years old. The anatomical position of implants was subglandular in 54.6% of cases and the remaining 45.4% was subpectoral.

The average breast thickness after compression was 56 mm varying between 7 and 102 mm and the average length of pectoral muscle was 32.1 mm varying between 0 and 141.6 mm in MLO and 66.5 mm ranging from 0 to 196.8 in ML projections.

#### Mammographic technique

Regarding technical aspects, the protocol followed by VD institutions was to perform 3 projections per breast (CC, MLO, and ML), while GVA institutions performed 2 projections (CC and MLO). This led to a total of 483 (40.0%) images in CC, 481 (39.9%) in MLO, and 243 (20.1%) in ML.

**Fig. 2:**
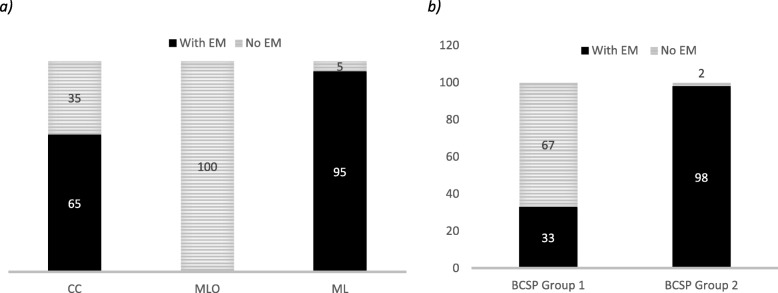
Eklund Manœuvre (EM): **a** percentage of exams (CC, craniocaudal; MLO, mediolateral oblique; and ML, mediolateral images) acquired with and without Eklund (EM); **b** EM according to each Regional Breast Cancer Screening Program (BCSP): Geneva (GVA) and Vaud (VD) for CC view

The Ecklund Manœuvre was performed in the majority of CC (64%) and ML (95%) images but MLO images were always acquired without using the Ecklund Manœuvre (Fig. 2a). Typically, the protocol in Vaud institutions included performing the Eklund Manœuvre (98%) while in Geneva the Manœuvre was performed only in 33% of exams (Fig. 2b).

The applied compression force varied between 0 and 188 Newton (N), the average compression with the Eklund Manœuvre was 38 ± 22.8 N and without was 56 ± 27.6 N.

The exposure parameters were determined selecting automatic (48.5%) or manual (51.5%) modes. Automatic mode for one manufacture was called the “Microdose” mode since it considers low dose strategies to acquire the images. The beam energy varied between 26 and 38 kVp and the equipment having the Microdose system always used higher energy (38 kVp).

#### Image quality criteria

**Fig. 3:**
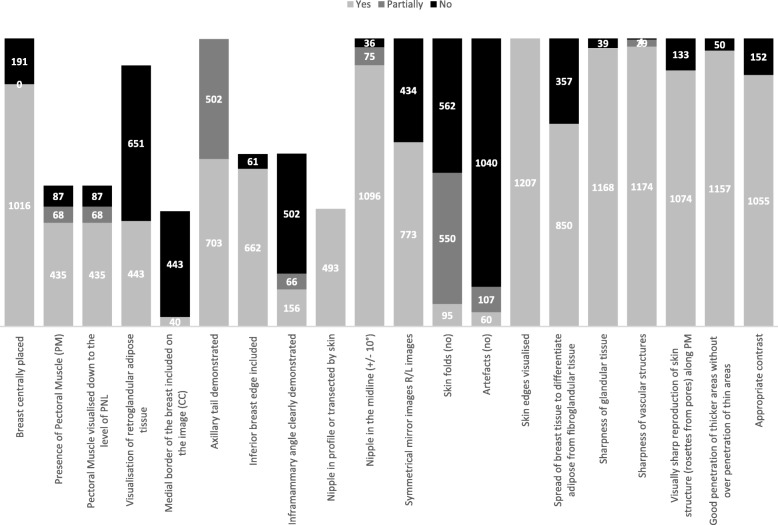
Image quality criteria assessed (total): yes, criterion observed; partially, some images did not achieve the criterion; no, criterion not observed

The breast was aligned to the detector for the majority of CC (92.5%), MLO (80.9%), and ML (74.1%) images but symmetry was not achieved in 37.9% of CC, 31.2% MLO, and 41.6% ML images. To assess these criteria, the observers assessed visually the distance between the centre of the breast and the limits of detector, laterally for CC and inferior/superior for ML and MLO images. If the distance was similar in both ways, it was considered centred; if not, it was considered as not centred. To verify if all glandular tissue is included in mammographic images, the visibility of retroglandular tissue was assessed and 50.5% of CC, 61.7% MLO, and 67.5% ML images did not present this anatomical area (Fig. 3). The visibility of posterior breast tissue was a criterion achieved in the majority of CC (70.3%) and MLO images (88.6%) but for ML images, it was more difficult (25.1%). The medial breast tissue was visible on 69.4% of CC images while the inferior edge was visible in 98.5% MLO and 77.7% of ML images (Fig. 3). However, if the breast is adequately centred/aligned to the detector, significant differences were observed for these parameters (*P* value = 0.001). When the breast was aligned, the visualisation of posterior breast tissue was better.

The criterion “pectoral muscle visible at least until to pectoral-to-nipple line (PNL)” was achieved in 40.1% of MLO and in 29.6% of ML images. The implant location (subglandular/subpectoral) seemed to have an impact on muscle length, existing differences (*p* < 0.0001) on breast tissue visualisation between both groups of images, being longer when the implant was subpectoral (Table 2). When the breast was centred over the detector, the inclusion of more muscle in the images was also observed, being noticed differences between images that were centred and the images non centred (*p* = 0.007).

**Table 2:** Impact of implant location (subglandular vs subpectoral) on pectoral muscle length in mediolateral oblique projections

Criterion	Level	Implant location	
		Subglandular	Subpectoral
Pectoral muscle inferior edge	At least nipple level	15 (7.1%)	141 (79.2%)
	Above superior edge of implant	181 (85.8%)	36 (20.2%)
	Short	11 (5.2%)	1 (0.6%)
	Absent	4 (1.9%)	0

**Fig. 4:**
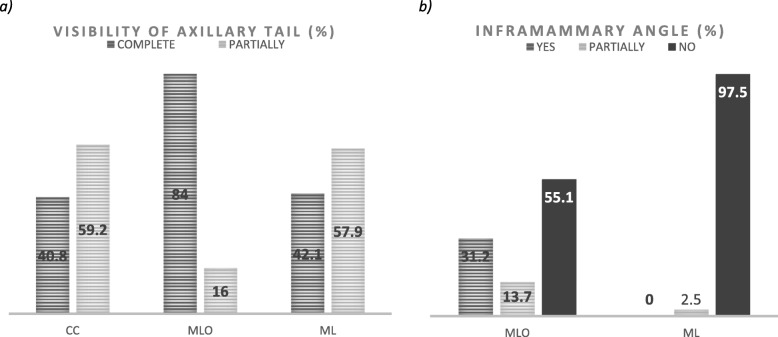
**a** Visibility of axillary tail visibility in CC, craniocaudal; MLO, mediolateral oblique; and ML, mediolateral images; **b** visibility of inframammary angle in MLO and ML images

The Axillary Tail (Spencer Tail) was visible in the majority of MLO images (84%), while in CC (59.2%) and ML (57.4%), it was not fully displayed (Fig. 4a). Significant differences were identified (*p* = 0.008) according to implant location, with the Axillary Tail more visible if the implant was retroglandular. The inframammary angle (IFMA) was absent in 97.5% of ML images and in 55.1% of MLO images (Fig. 4b). The nipple was visible in profile and in the midline (< 10°) for 89.6% of the images.

The majority of CC (93.2%), MLO (73.4%), and ML (97.5%) images did not present artefacts except minor skin folders (7.9%). However, the presence of a dark halo around the implant edge was noted making the breast tissue that was immediately in contact with the implant darker. The skin line was visible in all images but the visibility of rosettes from pores along the pectoral muscle was not clear/sharp.

Breast compression was not adequate to spread breast tissue in 51.6% (248/481) of images when breast was positioned for the MLO view, and 3.2% (38/1207) of images were blurry. The vascular structures were visible or partially visible in 99.7% of the examinations. The penetration of thicker areas without affecting the thin areas was adequate for 95.7% (1157/1207) images and contrast was also optimal for almost all examinations (87.4%) (Fig. 3).

Implants were included in 74.1% of CC, 99.6% of MLO, and 62.1% of ML images and the images were clear without blur. Applying the Eklund technique, the width of visible breast tissue in the images varied between 0.7 and 116.6 mm (Table 3). It was possible to observe a “gain” in the amount of tissue included when compared with the images acquired without this Manœuvre. CC images had a breast tissue gain of 8.9 ± 12.2 mm while ML had a gain of 9.6 ± 11.8 mm.

**Table 3:** Visible breast tissue without breast implant superimposition for all projections assessed

Projection	Visible breast tissue (mm)				
	N	Minimum	Maximum	Mean	Stde. Dev.
CC (craniocaudal)	483	3.6	108.2	43.9	19.5
MLO (mediolateral-oblique)	481	3.9	116.6	35.0	17.6
ML (mediolateral)	243	0.7	108.7	44.4	18.1

Analysing IQ globally, it was possible to verify that some criteria are dependent on implant location (subglandular or subpectoral) and also on the use of the Eklund Manœuvre. The main criteria dependent on the breast implant location and/or performing the Manœuvre were the visualisation of PM; retroglandular adipose tissue; posterior, medial, and superior tissues; and spread of glandular tissue (Table 4). The anterior implant edge inclusion seemed to be important since it helped to guarantee that all breast tissue was included in the image, demonstrating significant differences (*p* < 0.001) between the images with and without implant inclusion.

**Table 4:** Impact of implant position and Eklund Manœuvre on the achievement of image quality criteria (*p*, *p* value; *NS*, not significant)

Criteria	Implant position			Eklund Manœuvre		
	Eklund	No Eklund	Global	Subglandular implant	Subpectoral implant	Global
Breast centrally placed	*p* = < 0.01	NS	*p* = < 0.05	NS	NS	NS
Pectoral muscle (PM) visibility	*p* = < 0.001	*p* = < 0.001	*p* = < 0.001	NS	*p* = 0.002	NS
Visualisation of retroglandular adipose tissue	*p* = < 0.001	NS	*p* = < 0.001	*p* = < 0.001	*p* = < 0.001	*p* = < 0.001
Medial border of the breast included on the image	NS	*p* = 0.01	*p* = 0.05	*p* = < 0.001	NS	*p* = < 0.001
Axillary tail demonstrated	*p* = 0.01	NS	NS	*p* = < 0.001	NS	*p* = < 0.001
Nipple in the midline (+/− 10°)	NS	NS	NS	*p* = < 0.001	*p* = 0.01	*p* = < 0.001
Nipple in profile or transected by skin	NS	NS	NS	NS	NS	NS
No skin folds	*p* = < 0.001	NS	*p* = < 0.001	*p* = < 0.001	*p* = < 0.001	*p* = < 0.001
Spread of glandular tissue	NS	NS	*p* = 0.01	*p* = < 0.001	*p* = < 0.001	*p* = < 0.001
Sharpness	NS	NS	NS	NS	*p* = < 0.05	NS
Artefacts	*p* = 0.01	NS	NS	*p* = < 0.001	*p* = < 0.05	*p* = < 0.001
Symmetrical mirror images R/L images	*p* = 0.01	NS	*p* = < 0.001	*p* = < 0.001	*p* = 0.004	*p* = < 0.001
Beam penetration	NS	NS	NS	NS	NS	NS
Contrast	NS	NS	NS	NS	*p* = 0.002	*p* = < 0.05
Total of significant criteria	7	2	7	8	9	9

## Discussion

The main purpose of this study was to identify IQ criteria suitable for assessing mammographic examinations acquired in women with breast implants. The characterisation of BI mammography practice performed at two regional BCSP in Switzerland was also included in this study.

According to EUREF guidelines [6], radiographers are central to the success of mammography, having the responsibility of producing high quality mammograms. There are already several approaches available to evaluate each component of this chain [5] and IQ assessment is no exception. Image quality assessment is one of the most critical aspects for diagnosis since it combines many factors (equipment, positioning, technique, and radiographer-patient interaction). For that reason, it was not a surprise to verify that 22 parameters for assessing clinical IQ were identified in literature research [4, 47–50], with positioning being the larger group of items followed by artefacts, sharpness, and exposure parameters. Nevertheless, the published criteria and the recommendations provided by agencies and professional bodies do not cover IQ items to evaluate breast implants mammograms. The ACR, NHS from the UK and the British Society of Breast Radiology, Association of Breast Surgery Great Britain & Ireland and British Society of Plastic Reconstructive and Aesthetic Surgeons were the entities that provided guidelines for justification of clinical relevance of each medical imaging examination. The Eklund Manœuvre was also recommended by those entities as an essential technique to reduce implant overlap on breast tissue, facilitating image analysis and the diagnosis of breast pathologies [3, 19, 35]. However, it was not identified in the literature what compression force should be used, the exposure parameters suitable to breast density and thickness for BI, dose reference levels, how many projections should be performed, and criteria to assess image quality. This was reflected in the BCSPs participating in this study, with one group requiring the performance of 3 different positions (CC, MLO, ML) and another 2 (CC, MLO). There is no evidence that supports performing ML views (strict lateral) is beneficial to the patient providing additional information to the examination [19]. The balance between benefits and risks should be clearly identified to better define the protocol and to justify the practice, having in mind that performing 3 images will increase the radiation dose to the patient [23].

The Eklund Manœuvre is recommended by several screening programmes (UK, Australia, and USA) [19] because the implant displacement allows a better visualisation of breast tissue, although to assess the posterior breast tissue, the standard implant compression technique is considered more adequate [11, 22]. That means that both the displacement technique and standard implant compression technique should be performed to guarantee a complete study of breast tissue in patients with implants, but that has an impact on radiation dose doubling the value [11].

Even being recommended, Eklund technique was not always performed by the participants of this study and the literature presents several reasons for that, namely local protocols that not require it, the location of implant (with subpectoral location the superimposition of the implant is lower), or because the implant was encapsulated not allowing the Manœuvre [36]. The lack of confidence performing the Manœuvre and the short training received by radiographers were other reasons noted [10, 45]. It is suggested that the development of a training phantom would be beneficial to acquire the practical skills that are necessary to perform this Manœuvre.

Variations on compression force were identified but previous studies [51–53] showed the same variations in standard mammography examinations. Those studies highlighted that harmonisation in compression is necessary to minimise the problems related to dose, IQ, and pain management. Not compressing the breast can promote an increase on dose that can lead consequently to cell damage and it can also promote blur affecting the image quality. The images can also present blur when the compression is insufficient increasing the potential of having false-negative diagnosis since small or low-density lesions can be obscured.

The selection of exposure parameters in this specific context should consider the differences of the breast composition due to the presence of nonequivalent breast tissue which is denser and increases the beam energy and intensity [22, 54]. This change can be justified by the improvement of the equipment, because radiographers used automatic mode to image the breast when the Eklund Manœuvre was performed, avoiding the irradiation of this denser structure and gaining the appropriate exposure. Posteriorly, it was possible to use the same parameters on the manual mode when the implant was included.

The application of IQ criteria available in the literature for mammography without implants seemed to not always work for mammography performed on BI. Some criteria were not achieved due to the location of implants (subglandular/subpectoral) and that was also due to the selected technical approach (using the Eklund Manœuvre or not). The most obvious criteria not achieved were the visualisation of the pectoral muscle down to the level of the nipple, the visualisation of retroglandular adipose tissue and posterior glandular tissue, and the adequate spread of the breast. Considering subglandular implants, these results are to be expected since the implants overlap the anatomy if the Eklund technique is not performed. However, other aspects were explored and it was possible to verify that if the anterior border of BI was included in the image, a better visualisation of posterior tissues was achieved. There was a significant difference (*p* < 0.001) between the two groups of images, those with or without the anterior edge of the implant. If the degree of the implant included on the image was too large, a lesser result with regard to the spread of breast tissue was obtained. Despite that, it seems important to include the anterior edge of the implant but not the entire implant. Considering the results regarding the length of the pectoral muscle and its relationship to the nipple level, it shows that adjustments to the criterion are also necessary. For subglandular implants, the recommendation is to have the muscle just at the above superior edge of the implant, while for subpectoral muscle, the usual criterion (nipple level) can be applied. Nevertheless, a revision of positioning criteria is necessary because even for standard mammography, some items are open to a subjective interpretation, since some words such as “general amount of breast tissue” and “appropriate visualisation” are presented not being specific or measurable. Criteria concerning artefacts, sharpness, and exposure parameters noted for mammography can be applied to BI imaging.

The main limitations of this study are related to the lack of studies and guidelines dedicated to BI imaging quality analysis making a true systematic review or meta-analysis very difficult. Also, this study did not include all BCSP available in the country, not being possible to generalise these results. The number of observers participating was also reduced. However, a consensus was searched when different classifications (present, partially present, absent) were attributed to the criteria.

## Conclusions

Differences in protocols regarding the number of projections acquired, in technique (performing or not the Eklund Manœuvre), along with the use of manual or automatic modes were identified at the two breast cancer screening programmes in Switzerland included in this study. The analysis of image quality also revealed that the available criteria for mammography performed on patients without breast implants are not fully applicable to patients with implants. Adjustments are necessary, being important to consider the breast implant location and the technique. Visualising the anterior edge of the breast implant, and determining the pectoral muscle length analysis, alignment of the breast to the centre of the detector, and the degree of breast compression and artefacts can be considered to include in the list of criteria adapted to breast implants mammography assessment. However, it is also important to perform further studies to determine best practice regarding the amount of compression force/pressure, the appropriate number of projections, and the suitable exposure parameters, considering always the impact on radiation dose and image quality.

## Data Availability

Data generated or analysed during this study are included in this published article.

## Abbreviations

ACR: American College of Radiology
BCSP: Breast Cancer Screening Programs
BI: Breast implants
CC: Craniocaudal
DICOM: Digital Imaging and Communications in Medicine
GSDF: Greyscale standard display function
GVA: Geneva
IQ: Image quality
MLO: Mediolateral oblique
ML: Mediolateral
UK: United Kingdom
USA: United States of America
VD: Vaud
